# Asymmetric and parallel subgenome selection co-shape common carp domestication

**DOI:** 10.1186/s12915-023-01806-9

**Published:** 2024-01-02

**Authors:** Min Wang, Xinxin Li, Chongnv Wang, Ming Zou, Jing Yang, Xiang-dong Li, Baocheng Guo

**Affiliations:** 1 Key Laboratory of Zoological Systematics and Evolution, Institute of Zoology, Chinese Academy of Sciences, Beijing, 100101 China; 2 University of Chinese Academy of Sciences, Beijing, 100049 China; 3 Institute of Chinese Sturgeon, China Three Gorges Corporation, Yichang, 443100 Hubei China; 4 Hubei Key Laboratory of Three Gorges Project for Conservation of Fishes, Institute of Chinese Sturgeon, China Three Gorges Corporation, Yichang, 443100 Hubei China; 5 State Key Laboratory of Integrated Management of Insect Pests and Rodents, Institute of Zoology, Chinese Academy of Sciences, Beijing, 100101 China; 6 Center for Excellence in Animal Evolution and Genetics, Chinese Academy of Sciences, Kunming, 650223 Yunnan China; 7 Academy of Plateau Science and Sustainability, Qinghai Normal University, Xining, 810008 China

**Keywords:** Allopolyploid, Scale reduction, Vibrant skin color, High growth rate, Selection sweep

## Abstract

**Background:**

The common carp (*Cyprinus carpio*) might best represent the domesticated allopolyploid animals. Although subgenome divergence which is well-known to be a key to allopolyploid domestication has been comprehensively characterized in common carps, the link between genetic architecture underlying agronomic traits and subgenome divergence is unknown in the selective breeding of common carps globally.

**Results:**

We utilized a comprehensive SNP dataset in 13 representative common carp strains worldwide to detect genome-wide genetic variations associated with scale reduction, vibrant skin color, and high growth rate in common carp domestication. We identified numerous novel candidate genes underlie the three agronomically most desirable traits in domesticated common carps, providing potential molecular targets for future genetic improvement in the selective breeding of common carps. We found that independently selective breeding of the same agronomic trait (e.g., fast growing) in common carp domestication could result from completely different genetic variations, indicating the potential advantage of allopolyploid in domestication. We observed that candidate genes associated with scale reduction, vibrant skin color, and/or high growth rate are repeatedly enriched in the immune system, suggesting that domestication of common carps was often accompanied by the disease resistance improvement.

**Conclusions:**

In common carp domestication, asymmetric subgenome selection is prevalent, while parallel subgenome selection occurs in selective breeding of common carps. This observation is not due to asymmetric gene retention/loss between subgenomes but might be better explained by reduced pleiotropy through transposable element-mediated expression divergence between ohnologs. Our results demonstrate that domestication benefits from polyploidy not only in plants but also in animals.

**Supplementary Information:**

The online version contains supplementary material available at 10.1186/s12915-023-01806-9.

## Background

Many of well-known cultivated plants (e.g., maize, rice, soybean, wheat) are polyploid. Polyploidization or whole-genome duplication (WGD) is well acknowledged for providing new genetic materials that could enhance adaptability during plant domestication and their subsequent improvement [1]. For example, ohnologs — duplicated genes resulting from WGDs, are important in generating phenotypic novelties for agronomic design and the evolution of stress resistance [2–6]. In addition, convergent domestication of agronomic traits in an allopolyploid plant could result from genetic variations in a specific subgenome and/or both subgenomes [7–10]. Therefore, WGD is believed to be a critical factor in crop domestication [11–14].

As a matter of fact, polyploids are also frequently seen in aquaculture, and especially WGD is key to domestication in cyprinid carps [15]. The best-known cyprinid carp, common carp (*Cyprinus carpio*), is an evolutionary allotetraploid [16]. Common carp is among the earliest domesticated fishes, and its aquaculture in Neolithic China dates back 8000 years [17]. Nowadays, common carp is one of the most important farmed fishes in the global fishery and accounts for 7.7% (approximately 4.4 million tons) of the global freshwater aquaculture production [18]. Genetic basis underlying economically important traits (e.g., growth, disease resistance) in common carps has thus been extensively studied [19], mainly with quantitative trait locus (QTL) mapping approach as summarized in Chen et al. [20]. However, a single common carp strain is usually involved in most, if not all, of those QTL mapping studies. Meanwhile, common carp serves as an excellent model for studying the genome evolution of allopolyploids in vertebrates, and the divergence of evolutionary trajectories between the two subgenomes in common carp has been well characterized, especially the divergent evolution of ohnologs [21–24]. Although it is well-known that subgenome divergence is a key to allopolyploid domestication [25], the link between the genetic basis underlying domestication and subgenome divergence is unclear in common carp. Therefore, the genome-wide selection signatures underlying domestication while facing with subgenome divergence are largely unknown in the repeated selective breeding of common carps.

**Fig. 1:**
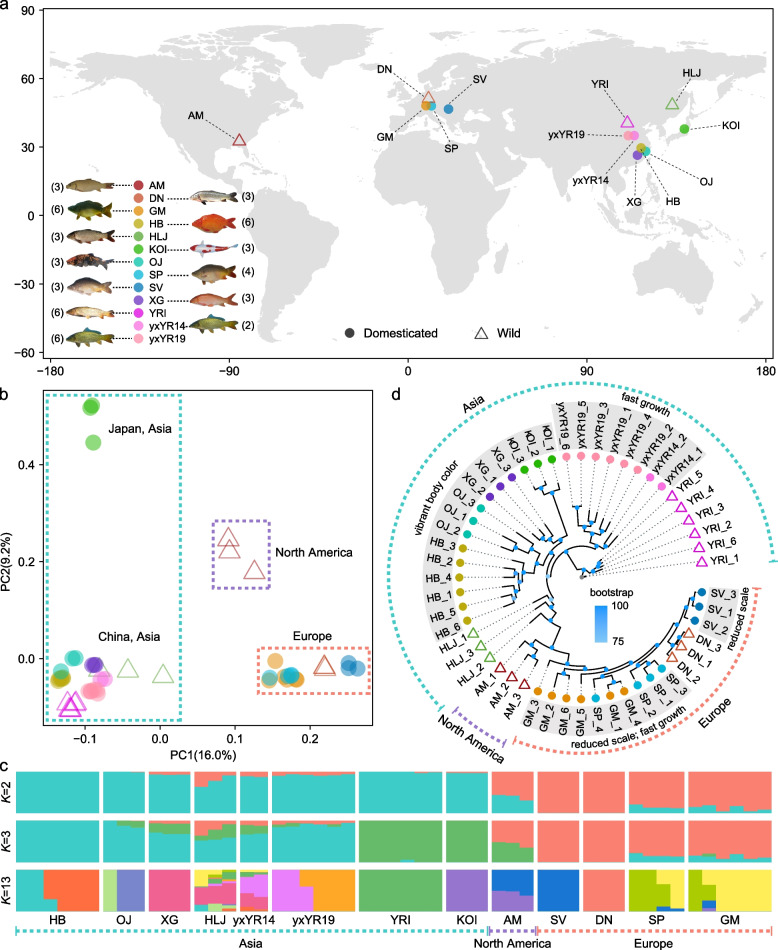
The population genetic structure of the 13 worldwide common carp strains based on 1.92 million genome-wide SNPs. **a** Sampling locations of common carps included in this study. Number in parentheses is number of individuals in each common carp strain (Additional file 2: Table S1). **b** Principal component analysis. **c** Bayesian model-based genetic clustering analysis. The number of populations (*K*) was predefined from 2 to 13, with the best-fit scenario of *K* = 2. **d** The maximum-likelihood phylogeny

In this study, we collected genomic data from 13 common carp strains globally (Fig. 1a; Additional file 2: Table S1) and utilized integrated population genetics approaches to detect genome-wide selective sweeps underlying three agronomically desirable traits — scale reduction, vibrant skin color, and high growth rate. Our results showed that asymmetrical subgenomic selection was prevalent in the domestication of the three agronomic traits, which was not attributable to the biased retention/loss of ohnologs between subgenomes but might be better explained by reduced pleiotropy through transposable element (TE)-mediated expression divergence between ohnologs, whereas parallel subgenomic selection is also observed in the skin-vibrant domesticated common carps. Taken together, our study demonstrates the advantage of expanded genetic degrees of freedom afforded by allopolyploid genome could have facilitated the domestication of common carps as well as plant domestication.

## Results

### Genetic diversity in common carps

A total of 51 common carp individuals from 13 globally representative strains, including eight strains (YRI, HLJ, yxYR19, yxYR14, HB, XG, OJ, and KOI) from Asia, four strains (DN, GM, SP, and SV) from Europe, and one strain (AM) from North America (Fig. 1a; Table S1), are included in this study. A total of 1,918,269 high-quality SNPs are identified, accounting for 1.347‰ of the common carp reference genome. With the genome-wide high-quality SNPs, two genetic clusters are consistently observed in the principal component and model-based population admixture analysis (Fig. 1b & c). These correspond to one cluster with common carps from Asia and another with common carps from Europe and North America. The KOI stain is distinct from other Asian strains according to the second principal component (Fig. 1b), and the HLJ strain from the northeast shows extensive genetic admixture with other strains (Fig. 1c; Additional file 1: Fig. S1). The North American strain is different from European strains according to the second principal component (Fig. 1b) and consisted of genetic components from both European and Asian common carps (possibly the SV and KOI strain from Asia and Europe, respectively; Fig. 1c) according to population admixture estimation. The genome-wide average differentiation of pairwise common carp strains (Additional file 1: Fig. S2d; Additional file 2: Table S2) is consistent with the above-mentioned observations. The maximum likelihood phylogeny inference supports repeatedly selection on high growth rate in independently selective breeding of common carps (Fig. 1d). In general, wild common carp strains have higher genome-wide average nucleotide diversity (*π*) than domesticated strains, and Asian strains higher than European strains, with the consideration of both sampling size and sequencing depth (Additional file 1: Fig. S2a; Additional file 2: Table S3). The genome-wide *π* and Tajima’ *D* are significantly different between subgenomes in most common carp strains (Additional file 1: Fig. S2a & b). The linkage disequilibrium (*LD*) decay in the domesticated strains with lower *π* is longer (Additional file 1: Fig. S2c).

### Genetic variation associated with scale reduction

**Fig. 2:**
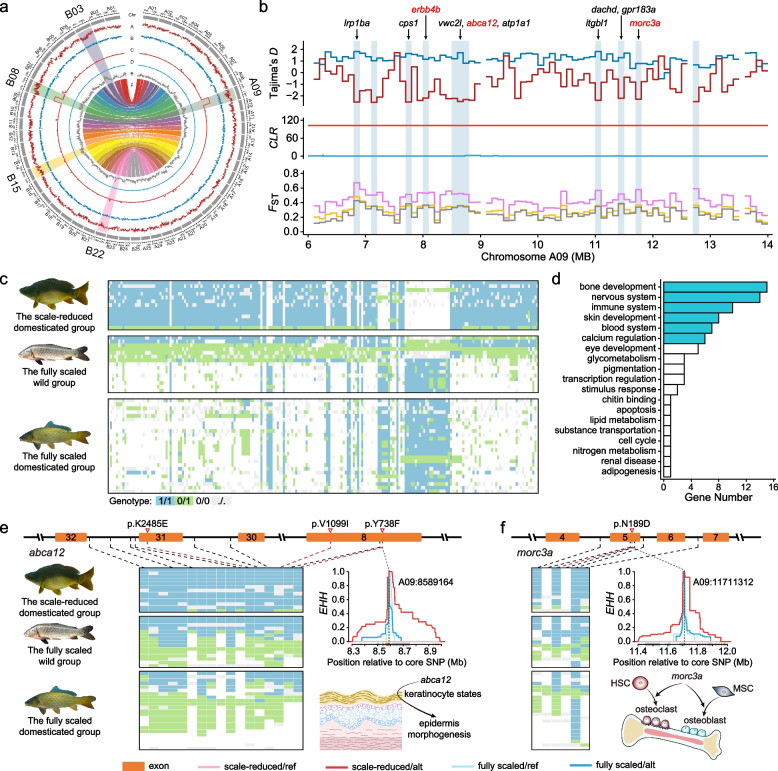
Genome-wide selection signatures associated with scale reduction in common carp domestication. **a** Genome-wide selection signals. Tracks A and B are Tajima’s *D* in the scale-reduced domesticated group and fully scaled wild group, respectively; Tracks C and D are *CLR* scores in the scale-reduced domesticated group and fully scaled wild group, respectively; Track E is *F*_ST_ between the scale-reduced domesticated group and fully scaled wild group; Track F is the synteny between subgenomes A and B. Chromosomes with signatures of selection are highlighted with larger font size of names (Additional file 2: Table S4). **b** Genomic regions with selection sweep signals on chromosome A09. Tajima’s *D* and *CLR* scores are calculated in the scale-reduced domesticated group (red lines) and the fully scaled wild group (blue lines), respectively. *F*_ST_ is calculated between the scale-reduced domesticated group and the fully scaled wild group (gray line), between the scale-reduced domesticated group and the fully scaled (wild and domesticated) group (orange line), and between the scale-reduced domesticated group and the fully scaled domesticated group (pink line). Light blue vertical bars with indicate the selection regions. Genes in the genomic regions (light blue vertical bars) with selection sweep signals are listed. **c** Genotypes of SNPs showing higher genetic differentiation between scale-reduced and fully scaled common carps in genomic regions with selection sweep signals on chromosome A09. **d** The GO in genomic regions with selection sweep signals related to scale reduction. **e** Genotypes of SNPs in the gene *abca12* and extended haplotype homozygosity (*EHH*) around the crucial SNP “A09:8573491” and “A09:8589164.” **f** Genotypes of SNPs in the gene *morc3a* and *EHH* around the crucial SNP “A09:11711312”

Scale-reduced strains (GM, SP, and SV; Additional file 2: Table S4) have been repeatedly selected in common carp domestication for consumption convenience [19, 20], although fish scales play important roles in mechanical protection and resistance to pathogenic microorganisms. A total of 1.90-Mb genomic regions harboring 2446 SNPs in chromosomes A09, B03, B08, B15, and B22 (Additional file 2: Table S5) show selection signatures associated with scale reduction in three domesticated common carp strains, with higher *CLR* scores (102.70) and negatively lower Tajima’s *D* values (−2.54 to −2.17) in the scale-reduced strains, as well as high *F*_ST_ values (0.33–0.49) between scale-reduced and fully scaled strains (Fig. 2a & b; Additional file 1: Fig. S3). Genotypes in scale-reduced strains obviously diverged from fully scaled strains (Fig. 2c). These results were repeatedly observed, when compared scale-reduced domesticated strains with fully scaled wild strains, fully scaled domesticated strains, and fully scaled wild and domesticated strains (Additional file 2: Table S5), respectively. Specially, the comparison between strains SV and DN might complement our results from pooling strains and be particularly informative to identify genetic variation associated with scale reduction, considering the closely phylogenetic relationship between SV and DN. As such, the comparison between strains SV and DN shows that the genomic regions harboring 1459 of 2446 SNPs in chromosomes A09 and B03 abovementioned might be particularly associated with scale reduction.

In the 1.90-Mb genomic regions with selection signatures, 56 genes are found, and they are involved in development and metabolism, especially bone development, skin development, calcium regulation, nervous system, and immunity (Fig. 2d; Additional file 2: Table S6). Although the potentially causative gene — fibroblast growth factor receptor 1a1 (*fgfr1a1*) identified in earlier studies [21, 26, 27], is not located in the genomic regions with selection signatures here, genes involved in bone and/or skin development are indeed frequently observed (Fig. 2d), and several of them might play a pivotal role in scale reduction in domesticated common carps. The gene *ATP-binding cassette subfamily A member 12* (*abca12*) is essential for keratinocyte organization in epidermis morphogenesis in zebrafish [28]. Three nonsynonymous mutations in *abca12* are found in the scale-reduced common carp strains, two of which (A09:8573491 and A09:8589164) are radical substitutions and lead to replacement of amino acid with different physicochemical properties (Fig. 2e). The extended haplotype homozygosity (*EHH*) values decline gradually around these three nonsynonymous mutations in *abca12* in scale-reduced common carp strains but sharply in fully scaled common carp strains (Fig. 2e), suggesting strong selection on these three nonsynonymous mutations. Similar results are also observed in gene *MORC family CW-type zinc finger 3a* (*morc3a*, Fig. 2f) that is the regulator of cortical bone homeostasis by involving in differentiation of osteoblast and steoclast [29] and gene *erb-b2 receptor tyrosine kinase 4b* (*erbb4b*, Additional file 1: Fig. S4a) — a paralog of *erbb3b* that is a key gene in scale formation in zebrafish [30]. The gene *tripartite motif containing 33* (*trim33*) which is essential for not only osteoblast proliferation and differentiation via the bone morphogenetic protein pathway [31] but also plays a significant role in innate immune regulation in zebrafish [32] harbors nonsynonymous mutation under strong selection in the scale-reduced common carp strains (Additional file 1: Fig. S4b), as genes involved in bone and/or skin development above-mentioned. TRIM33 protein degrades the antiviral protein viperin_sv1 to promote replication of spring viremia of carp (SVC) virus [33] — a virus that has resulted in significant morbidity and mortality in European common carp culture [34, 35]. The missense mutation may alter the interaction and colocalization of *trim33* with viperin_sv1 protein and further protect scale-reduced common carps from SVC infection. The enhancement of the internal disease resistance through such genetic variation in *trim33* might compensate for the decrease of physical immune defense due to scale reduction in scale-reduced domesticated common carps, which might play a key role in the establishment of scale-reduced strains.

### Genetic variation associated with vibrant skin color

**Fig. 3:**
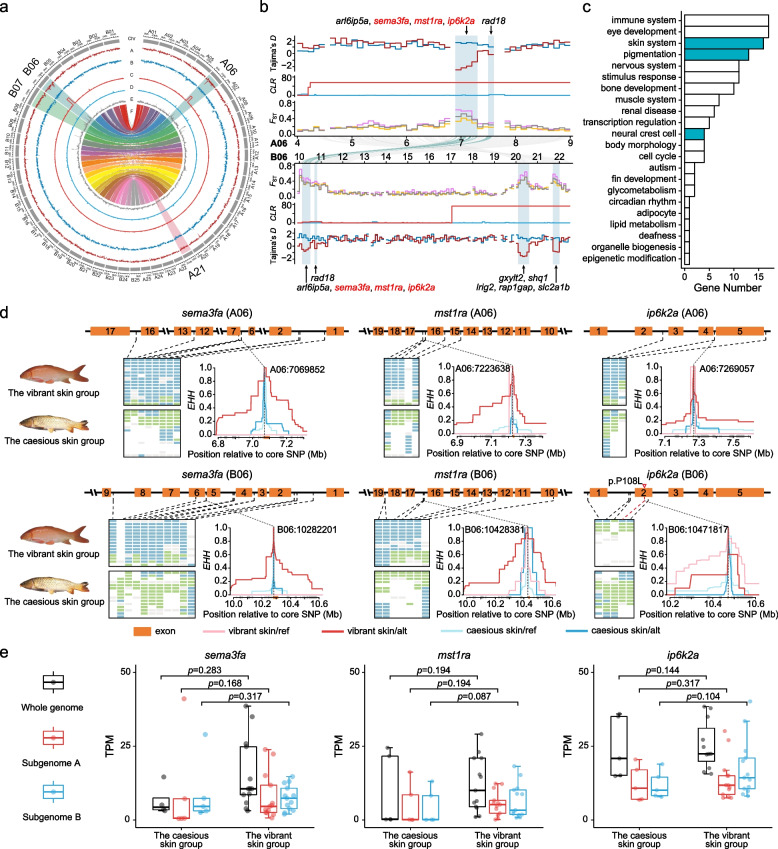
Genome-wide selection signatures associated with skin color variation in common carp domestication. **a** Genome-wide selection signals. Tracks A and B are Tajima’s *D* in the skin-vibrant domesticated group and the skin-caesious wild group, respectively; Tracks C and D are *CLR* scores in the skin-vibrant domesticated group and the skin-caesious wild group, respectively; Track E is *F*_ST_ between the skin-vibrant domesticated group and the skin-caesious wild group; Track F is the synteny between subgenomes A and B. Chromosomes with signatures of selection are highlighted with larger font size of names (Additional file 2: Table S4). **b** Genomic regions with selection sweep signals on chromosomes of A06 and B06. Tajima’s *D* and *CLR* scores are calculated in the skin-vibrant domesticated group (red lines) and the skin-caesious wild group (blue lines), respectively. *F*_ST_ is calculated between the skin-vibrant domesticated group and the skin-caesious wild group (gray line), between the skin-vibrant domesticated group and the skin-caesious (wild and domesticated) group (orange line), and between the skin-vibrant group and the skin-caesious Asian wild group (pink line). Light blue vertical bars which indicate the selection regions. Genes in the genomic regions (light blue vertical bars) with selection sweep signals are listed. Lines between chromosomes of A06 and B06 show synteny between the two paralogous chromosomes. **c** The GO in genomic regions with selection sweep signals related to skin color variation. **d** Genotypes of SNPs in three pairs of paralogous genes on A06 and B06 and extended haplotype homozygosity around the crucial SNPs in each of the six genes. **e** Expression in skin of the three pairs of ohnologs with selection signals associated with color variation in common carps (Additional file 2: Table S10)

Colored varieties of common carps are used for ornamental purposes globally, especially in China and Japan. The four skin-vibrant domesticated strains (HB, XG, OJ, and KOI) form a monophyletic clade distinct from other strains (Fig. 1d) suggesting that they might be derived from a founder population and have shared genetic variation associated with their vibrant skin. By comparing with skin-caesious wild and/or domesticated strains (Additional file 2: Table S4), a total of 2.04-Mb genomic regions in chromosomes of A06, B06, B07, and A21 are found to consistently show selection signatures associated with vibrant skin color signals (Fig. 3a & b; Additional file 1: Fig. S5). The 2.04-Mb genomic regions with 2503 SNPs contain 77 genes, many of which are involved in pigmentation, neural crest cell development, skin disease, and immunity (Fig. 3c; Additional file 2: Tables S7 & S8). There are 1.04-Mb genomic regions on chromosomes A21, B06, and B07 showing asymmetric selection signatures associated with vibrant skin color signals (Fig. 3a & b), harboring 31 genes (Table S7), many of which are related to pigmentation. For example, zebrafish *slc2a1b* morphants display less pigmentation [36]; *cpeb4b*, *lrig2*, and *rap1gap* regulate the proliferation and survival of melanoma cells [37–39]; and *gxylt2*, *shq1*, and *rhcga* are related to skin coloring diseases in human [40–42].

Interestingly, a pair of 0.5-Mb homologous genomic regions between chromosome A06 and B06 shows selection signatures associated with vibrant skin color signals (Fig. 3a & b), harboring 18 pairs of ohnologs (Table S7). Five out of the 18 pairs of ohnologs are involved in pigmentation (Table S8), and three-pair ohnologs of *mst1ra* (*macrophage-stimulating 1 receptor a*), *sema3fa* (*sema domain*, *immunoglobulin domain [Ig]*, *short basic domain*, *secreted*, *[semaphorin] 3Fa*), and *ip6k2a* (*inositol hexakisphosphate kinase 2a*) show strong selection signals and divergent genotypes between the skin-vibrant and skin-caesious group (Fig. 3b & d). The *mst1ra* is involved in melanoma development [43], and zebrafish knockout mutants of *sema3fa* or *ip6k2a* show disrupted development and migration of neural crest cells, a kind of stem cells finally differentiated into different functional cell lines including pigment cells [44–46]. The *EHH* values from the core loci in each gene of the three-pair ohnologs are much higher in the skin-vibrant group than those in the skin-caesious group, suggesting parallel selection on both copies in each of the three pair ohnologs (Fig. 3d). RNA-seq data (Additional file 2: Table S9) show that both copies in each of the five-pair ohnologs involved in pigmentation are expressed in common carp skin, with higher expression in skin-vibrant group than that in skin-caesious group (*P* = 0.072–0.317, Wilcoxon signed-rank tests; Fig. 3e; Additional file 1: Fig. S6; Additional file 2: Table S10). Our results show that parallel selection on ohnologs in both subgenomes, together with asymmetric selection on genes in specific subgenome, underlies skin-vibrant domesticated common carp selection.

### Genetic variation associated with high growth rate

**Fig. 4:**
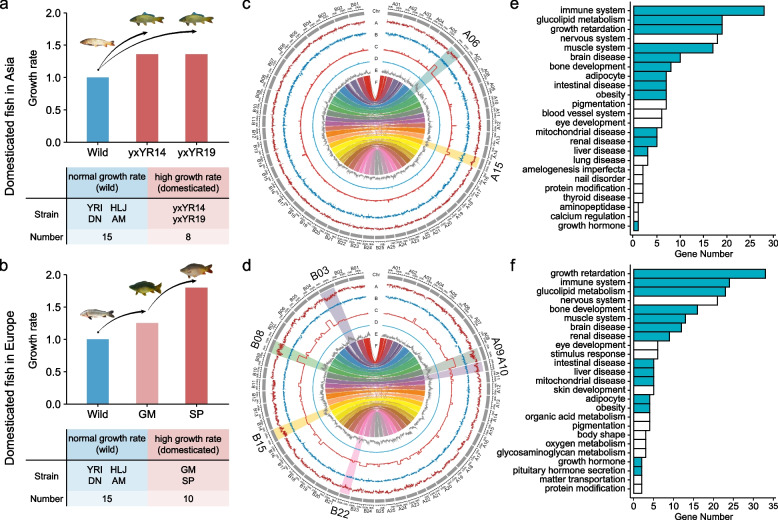
Genome-wide selection signatures associated with high growth rate in common carp domestication. Growth rate in wild and domesticated common carps from Asia (**a**) and Europe (**b**). Genome-wide selection signatures associated with high growth rate in domesticated common carps from Asia (**c**) and Europe (**d**). Tracks A and B are Tajima’s *D* in the high growth rate domesticated and wild group; Tracks C and D are *CLR* score in the high growth rate domesticated and wild group; Track E is *F*_ST_ between the high growth rate domesticated and wild group; Track F is the synteny between subgenome A and B (Additional file 2: Table S4). Chromosomes with signatures of selection are highlighted with larger font size of names. The GO in genomic regions with selection sweep signals related to high growth rate in domesticated common carps from Asia (**e**) and Europe (**f**)

High growth rate is the primarily agronomical trait in common carp breeding. Fast-growing common carps have been selectively bred from wild strains in Asia and Europe, respectively. The growth rate in Yuxuan Yellow River carp (e.g., yxYR14 and yxYR19 strains) is 30% faster than that in wild strains [47, 48] (Fig. 4a). The growth rate in strains GM and SP selected from the European wild strains increases 20–30% and 80% compared with wild common carps [48–50], respectively (Fig. 4b). Population genetic comparisons between fast-growing domesticated strains and wild strains (Fig. 4a & b; Additional file 2: Table S4) identify 3.3-Mb genomic regions with 4749 SNPs and 121 genes in chromosomes A06 and A15 (Fig. 4c; Additional file 1: Fig. S7; Additional file 2: Table S11) and other 3.2-Mb genomic regions with 4803 SNPs and 145 genes in chromosomes A09, A10, B03, B08, B15, and B22 (Fig. 4d; Additional file 1: Fig. S7; Additional file 2: Table S11) associated with selective breeding of fast-growing domesticated strains from Asian and European wild strains, respectively. Although neither genomic regions nor genes associated with high growth rate are shared between Asian and European fast-growing domesticated strains, those genes are involved in same GOs (Fig. 4e & f; Additional file 2: Table S12). Our results highlight that selection on metabolism process (e.g., glucolipid, organic acid, oxygen), development process (e.g., bone, muscle, nerve, immune), and anti-disease (e.g., growth retardation, obesity, renal, liver) collectively contribute to fast growing in common carp breeding.

### Divergence between ohnologs associated with common carp domestication

**Fig. 5:**
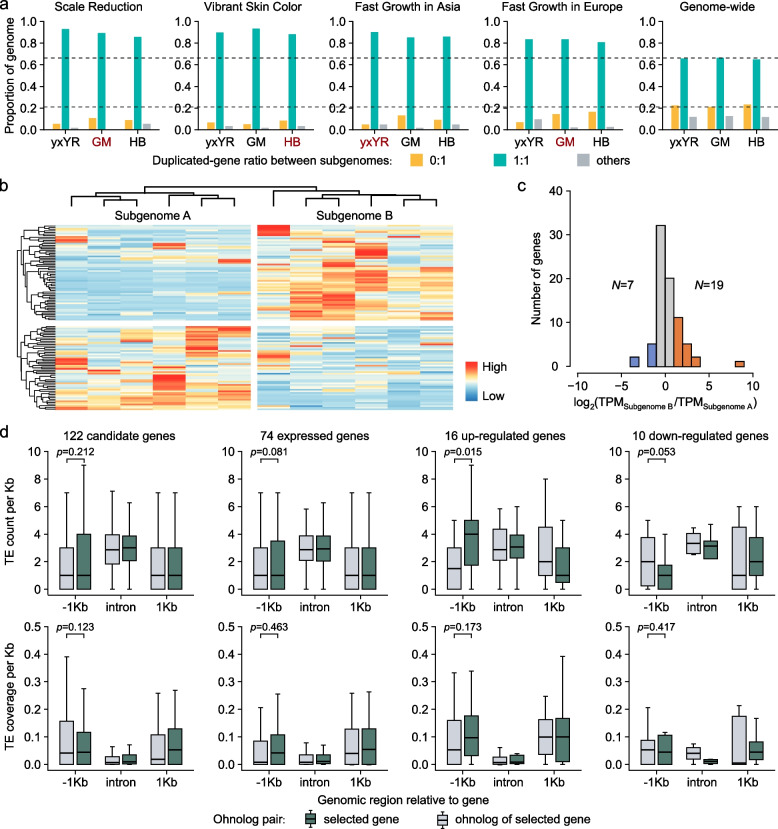
Ohnolog dynamics in genomic regions with selection sweep signals in common carp genomes. **a** Ohnolog dynamics in genomic regions with selection sweep signals related to scale reduction, skin color variation, and fast growth and genome-wide ohnolog dynamics in common carp genomes. The ratios of 1:0, 1:1, and others represent singleton genes, ohnologs, and multiple-copy genes in the genome of Yuxuan Yellow River carp (yxYR), German mirror carp (GM), and Hebao red carp (HB), respectively. **b** Expression of 121 ohnolog pairs between subgenomes in the muscle of the strain SP (Additional file 2: Table S15). **c** Ohnolog with twofold expression divergence. Expression divergence between 78 pairs of expressed ohnologs with *TPM* > 1 in at least one sample in the muscle of the strain SP. Log_2_(TPM_Subgenome B_/TPM_Subgenome A_) indicates the degree of expression difference of the ohnolog pairs. *N* values indicate the number of ohnolog pairs with twofold expression divergence. **d** TE content and coverage in gene body (intron), upstream (1 Kb), and downstream (1 Kb) regions between the 122 pairs of ohnologs, 74 pairs of expressed ohnologs, 16 pairs of ohnologs with twofold upregulated expression in selected genes, and 10 pairs of ohnologs with twofold down-regulated expression in selected genes in GM genome, respectively

To investigate if asymmetric subgenome selection results from biased retention/loss of one gene in ohnologs, we examined the gene retention and loss between subgenomes in strain yxYR, GM, and HB, respectively. Our result shows that the gene loss ratio in genomic regions with selection signatures associated with scale reduced, skin vibrant, and/or fast growing (0.05–0.17) is significantly lower (*χ*^2^ tests, *P* < 9.2 × 10^−16^; Fig. 5a; Additional file 2: Table S13) than that genome wide (0.21–0.23). In 363 of the 381 ohnolog pairs harbored in genomic regions with asymmetric selection signatures, both copies in 262 ohnolog pairs are found to be retained in all of the three common carp yxYR, HB, and GM genomes (Additional file 2: Table S14). It suggests that the asymmetric subgenome selection in common carp domestication does not seem to be associated with biased retention/loss of ohnologs in genomic regions with selection signatures associated with domestication.

Next, we analyzed gene expression in 121 one-to-one ohnolog pairs with genes associated with the fast growth of European common carps in SP strain with available transcriptomic data from muscle (Additional file 2: Table S9), since biased gene expression is known to be prevalent between subgenomes in common carps [22, 24]. We found that 26 ohnolog pairs showed twofold expression divergence (Fig. 5b & c; Table S15). Among the 26 ohnolog pairs, 16 genes with selection signal are significantly upregulated (*P* = 0.016, Wilcoxon signed-rank tests; Additional file 1: Fig. S8a–p), and 10 with selection signal are significantly downregulated to their ohnologs without selection signal (*P* = 0.016, Wilcoxon signed-rank tests; Additional file 1: Fig. S8q–z).

Finally, TEs (Fig. 5d; Additional file 1: Fig. S9) and genetic polymorphism (Additional file 1: Fig. S10) in 1-Kb upstream region, intron region, and 1-Kb downstream region of the 122 one-to-one ohnolog pairs associated with fast growth in European common carps were investigated to understand their expression divergence. We observed that the number of TE rather than TE coverage or genetic polymorphism (Fig. 5d; Additional file 2: Table S16; Additional file 1: Fig. S10) is significantly increased in 1-Kb upstream region of the selected genes showing twofold upregulated expression (*P* = 0.015, Wilcoxon signed-rank test) and decreased in 1-Kb upstream region of the selected genes showing twofold down-regulated expression compared to their ohnologs (*P* = 0.053, Wilcoxon signed-rank test). Specially, we found that the number of DNA transposons from superfamily DTC was significantly increased in 1-Kb upstream of the up-regulated genes with selection signal (*P* = 0.032, Wilcoxon signed-rank test; Additional file 1: Fig. S9). Taken together, our results demonstrated that TE-mediated expression divergence between ohnologs might explain asymmetric subgenome selection in common carp domestication.

## Discussion

Deciphering genetic architecture underlying agronomic traits is key to genetic improvement in future common carp domestication. Using genome-wide SNP data from a representative sampling of common carps, we identified genome-wide genetic variations associated with the selective breeding of common carps. Many genes which have not been identified by earlier studies [20] are found to be associated with scale reduction, vibrant skin color, and high growth rate in domesticated common carps (Figs. 2, 3 and 4), respectively. Notably, no selection signature is detected in previously known potentially causative gene *fgfr1* for scale reduction in common carps [21, 26, 27], suggesting that the independent scale reduction in common carps might result from different gene variations. In the meanwhile, 31 genes are found in 1.2-Mb genomic regions associated with both scale reduction and high growth rate, both of which are simultaneously and directionally selected traits in domesticated mirror carps [51], reflecting the genetic correlation between agronomic traits in common carps. While the genetic basis underlying scale reduction, vibrant skin color, and/or high growth rate themselves are interesting to be known [21, 26, 27], our results also provide insights into the genetic architecture of other important target traits in the selective breeding of common carps. Genes associated with scale reduction, vibrant skin color, and high growth rate are found to be repeatedly enriched in the immune system (Figs. 2d, 3c, 4e & f), which indicates that the selective breeding of scale-reduced, skin-vibrant, and/or fast-growing common carps is accompanied by improved disease resistance. It is well-known that fish scales play important roles in mechanical protection and resistance to pathogenic microorganisms, and fully scaled common carps are more resistant to white spot disease than scale-reduced common carps [52]. Thus, scale-reduced domesticated common carps have meanwhile been selected for improved disease resistance [51]. Therefore, our findings provide potentially novel molecular targets not only for future genetic improvement in the selective breeding of scale-reduced, skin-vibrant, and/or fast-growing common carps but also for developing therapeutic strategies to halt viral infection in common carp culture (e.g., *trim33* gene, Additional file 1: Fig. S4).

The outcomes of domestication are shaped by artificial selection, which could occur on genetic variations in either one of subgenomes — asymmetric subgenome selection [8, 10] or homoeologous regions between subgenomes — parallel subgenome selection [7] in allopolyploid domestication. By investigating selection imprints in the selective breeding of scale-reduced, skin-vibrant, and/or fast-growing common carps, we find that asymmetric subgenome selection is prevalent in common carp domestication. The prevalence of asymmetric subgenome selection in common carp domestication does not seem to be associated with biased retention/loss of ohnologs in genomic regions with selection signatures associated with domestication between subgenomes, although asymmetrical gene retention/loss occur between subgenomes in common carps [21–24]. In contrast, the crosstalk between subfunctionalization in ohnologs and pervasive pleiotropy in domestication of complex traits [53, 54] might better explain the prevalence of asymmetric subgenome selection in common carp domestication, since subfunctionalization through expression divergence occurs rapidly between ohnologs after WGD [55]. We indeed observed extensive expression divergence between one-to-one ohnologs related to the fast growth of European common carps, which might result from TE content changes in upstream and downstream regions between genes with and without selection in ohnolog pairs (Fig. 5d). In fact, TE are known to be essential elements in gene expression regulation [56], and polyploidization could induce TE activity and generate a wide variety of changes in gene expression, which might explain part of the new phenotypes observed and contribute to the domestication of polyploid plants [57, 58]. Asymmetric expression is well-known in common carps [21–24], and it is thus not surprising that independently selective breeding of fast-growing common carps has adopted completely different genetic changes (Fig. 4). It says that selection on genetic variations in one subgenome could result in agronomically desirable trait, and evolutionary constraint due to pleiotropy could be reduced in allopolyploid domestication compared to that in diploids. While asymmetric subgenome selection is prevalent, parallel subgenome selection occurs in the skin-vibrant common carp breeding (Fig. 3). The parallel subgenome selection in the skin-vibrant common carp breeding might suggest that after WGD dosage, balance is required in the genetic regulatory network of trait (viz., vibrant skin) development [59], since both copies of ohnolog with a selective signal are highly expressed in skin-vibrant common carp skin (Fig. 3e; Additional file 1: Fig. S6; Additional file 2: Table S10). Taken together, our findings show that genetic architecture underlying agronomic traits in common carp domestication is shaped by not only the prevalently asymmetric but also parallel subgenome selection.

## Conclusions

Our comprehensive genomic scan across a representative sampling of common carps globally detects genome-wide genetic variations associated with the selective breeding of scale-reduced, skin-vibrant, and/or fast-growing common carps. In common carp domestication, asymmetric subgenome selection is prevalent, while parallel subgenome selection occurs, which is not due to asymmetric gene retention/loss between subgenomes but might be better explained by reduced pleiotropy through TE-mediated expression divergence between ohnologs after WGD. Overall, our results demonstrate that domestication benefits from polyploidy not only in plants but also in animals.

## Methods

### Sampling, sequencing, and data collection

Whole-genome re-sequencing data of 51 common carp individuals representing globally 4 wild and 9 domesticated strains was collected (Fig. 1; Additional file 2: Table S1). Six individuals from Hohhot in Inner Mongolia (YRI) were whole-genome re-sequenced to represent wild common carps from the Yellow River drainage. Genomic DNA extraction, DNA library construction, and sequencing were done by Annoroad Gene Technology Beijing Co. Ltd. Briefly, genomic DNA was extracted from ethanol-preserved fin clips; DNA library with an insert size of 300–500 bp was constructed for each individual and sequenced on the Illumina HiSeq2000 platform with a 150-bp paired-end strategy [60]. Whole genome re-sequencing data of nine individuals from the three distinct wild strains (HLJ, AM, and DN) and 36 individuals from nine domesticated strains (yxYR14, yxYR19, HB, XG, OJ, KOI, GM, SP, and SV) was retrieved from the GenBank sequence database [61, 62]. As such, a total of 1056-Gb whole-genome re-sequencing data was involved in this study, which results in 5–16 × coverage of the common carp genome [22] in each individual for single-nucleotide polymorphisms (SNPs) identification. Detailed information on the individuals sampled for genetic variation analysis in this study is given in Additional file 2: Table S1. In addition, 150-Gb skin transcriptomic data of 23 common carp individuals from 6 strains were retrieved from the GenBank sequence database for gene expression variation comparison between the vibrant and caesious skin common carps [62–67], as well as 49-Gb muscle transcriptomic data of six common carp individuals from the strain SP for expression variation comparison between subgenomes (Additional file 2: Table S9) [67, 68]. The Yuxuan Yellow River carp genome (GenBank assembly accession: GCA_004011575.1) [22, 62] with two determined subgenomes was retrieved as the reference genome for the following read mapping and gene annotation.

### Variant calling, filtering, and annotation

The raw reads from whole-genome re-sequencing were trimmed using Trimmomatic v0.39 [69] with the following parameters, ILLUMINACLIP:TruSeq3-PE.fa:2:30:10, SLIDINGWINDOW:4:20, LEADING:3, TRAILING:3, and MINLEN:50, and further quality checked using FastQC v0.11.9 [70]. Duplicates were removed using FastUniq v1.1 [71] with default parameters. Quality filtered reads were mapped to the reference genome using BWA-MEM v0.7.17 [72] with default parameters. The mapping rate of the sequencing reads to the reference genome ranged between 98.82 and 99.46% within each individual. The mapping results in SAM format were converted from into BAM format and then sorted according to mapping coordinates using SAMTools v1.9 [73]. The putative PCR-generated duplicated read pairs were marked using the MarkDuplicatesSpark function in Genome Analysis Toolkit (GATK) v4.1.8.1 [74].

Genomic variants in genomic variant call format (GVCF) for each individual were identified using the HaplotypeCaller module and the GVCF model in GATK. All of the GVCF files were then merged into a single vcf file. To remove the potential false positives, variants were filtered as follows: (1) removing SNPs within 10 bp of an indel using BCFTools v1.9 [73], (2) excluding indels using VCFTools v0.1.16 [75], (3) further filtering using VCFtools with the parameters of “--minQ 30 --min-alleles 2 --max-alleles 2 --minDP 2 --maxDP 40 --minGQ 20 --max-missing 0.5 --maf 0.05”, and (4) final filtering using VariantFiltration function in GATK with the parameters of “QD < 5.0 || FS > 30.0 || MQ < 50.0 || SOR > 3.0 || MQRankSum < -5.0 || ReadPosRankSum < -5.0.” A total of 1.92 million high-quality SNPs was finally determined for the following analyses, which accounted for 1.35‰ of the reference genome.

SNPs annotation was performed based on the Yuxuan Yellow River carp genome using ANNOVAR v2020-06-07 [76]. Among the 1.92 million high-quality SNPs, 9.91% (190,638), 48.49% (930,075), 6.87% (131,796), 0.02% (358), and 34.69% (665,403) were located in exonic, intronic, upstream/downstream (1-Kb flanking regions of a gene), splicing, and intergenic regions, respectively. SNPs in the coding regions, 62,901 and 116,038 were nonsynonymous and synonymous substitutions, respectively.

### Population genetic analyses

The genome-wide population genetic parameters, nucleotide diversity (*π*), Tajima’s *D*, and pairwise fixation index (*F*_ST_) were calculated using VCFTools v0.1.16 with a 100-Kb non-overlapping sliding window. The signification of mean value difference (*π* and Tajima’s *D*) between the two subgenomes was compared by permutation test using the R package Deducer [77]. The squared correlation coefficient between SNP pairs (*r*^2^) was estimated using PopLDdecay [78] to measure linkage disequilibrium. A maximum likelihood phylogeny was constructed using IQ-TREE v2.1.2 [79] with automatically selected best-fitting model and 1000 ultrafast bootstrap replicates. Principal components analysis (PCA) was conducted using PLINK v1.9 [80]. The population genetic cluster inference was performed using ADMIXTURE v1.3.0 [81] with the 1.92 million high-quality SNPs being filtered using PLINK with the parameters of “--geno 0.05 --hwe 0.0001.” ADMIXTURE was run with the presumptive population number (*K* value) ranging from 1 to 15 and the option of “--cv” for cross-validation to identify the best *K* value.

### Selection signals detection

To investigate genome-wide selection signatures, three population genetic parameters, site frequency spectra (SFS), Tajima’s *D*, and *F*_ST_ were collectively utilized. Genome-wide selective sweeps related to a specific trait domestication (reduced scale, vibrant skin color, or high growth rate) in relevant domesticated strains (Additional file 2: Table S4) were detected according to both SFS estimated using the composite likelihood ratio (*CLR*) test in SweeD v4.0.0 [82] with approximately 100-Kb window through the reference genome and Tajima’s *D* calculated in 100-Kb nonoverlapping sliding windows using VCFTools. Genome-wide genetic differentiation was estimated between domesticated strains with a specific trait (reduced scale, vibrant skin color, or high growth rate) and (wild and/or domesticated) strains without the specific trait by calculating *F*_ST_ values in 100-Kb nonoverlapping sliding windows using VCFTools. Only genomic regions with the top 1% *CLR* scores, the bottom 1% Tajima’s *D* values, and top 1% *F*_ST_ values were considered as candidates experienced selective sweeps related to a specific trait domestication. The extended haplotype homozygosity (*EHH*) approach was adopted to validate signature of selection in candidate SNPs using the R package of rehh [83].

### Ortholog identification

The orthology between common carp and zebrafish genome (GRCz11) were obtained with all-against-all blast using BLASTP v2.5.0 [84] with e-value <= 1e-10. Homoeologous gene and pairwise collinearity between subgenomes in the reference common carp genome were identified using MCScanX [85], in which only the five best syntenic blocks between chromosome pairs were reserved. Orthologous genes among the three common carp genomes, yxYR (GCA_004011575.1), GM (GCA_004011555.1), and HB (GCA_004011595.1) [22, 62], were identified using OrthoFinder v2.5.2 [86]. Pearson’s chi-square test with correction was used for testing the distribution difference of paralogue gene pairs in subgenomes between selection regions and the whole genome. Gene Ontology (GO) and Kyoto Encyclopedia of Genes and Genomes (KEGG) pathway annotations were based on the orthology between common carp and zebrafish genome. GO and KEGG pathway enrichment analyses were conducted for genes in genomic regions under selection using the R package of topGO [87] with Fisher exact tests and Bonferroni correction for false discovery rate correction.

### Gene expression quantification

Raw reads from skin or muscle transcriptomes were filtered by FASTP v0.20.1 [88] with default parameters to exclude reads with low quality. Quality filtered reads then were mapped to the reference genome using HISAT2 v2.1.0 [89]. Gene expression was quantified and normalized to transcripts per million (TPM) value using StringTie v2.1.4 [90]. Wilcoxon signed-rank test was performed to test the significance of gene expression divergence between common carp groups as well as one-to-one onhologs between subgenomes.

### Transposable element identification

We predicted TEs and constructed a nonredundant TE library for GM genome using Extensive *de novo* TE Annotator (EDTA) v2.1.3 [91] by allowing RepeatModeler to identify novol TEs. Then, the total TE content was identified using RepeatMasker v4.1.1 [92] based on the constructed TE library. TE content was further compared between ohnologs in the gene body (intron), upstream (within one Kb), and downstream (within 1 Kb) region using Wilcoxon signed-rank tests.

### Supplementary Information

**Additional file 1:** **Fig. S1.** Bayesian model–based clustering analysis for 51 common carp individuals. **Fig. S2.** Population genetic parameter estimation. **Fig. S3.** Selection signals related to scale reduction. **Fig. S4.** Haplotype analyses in gene erbb4b and trim33 related to scale reduction. **Fig. S5.** Selection signals related to vibrant skin color. **Fig. S6.** Gene expression of the two parallel selected ohnolog pairs. **Fig. S7.** The putative selection sweep signals related to high growth rate. **Fig. S8.** Expression divergence between one-to-one ohnologs including genes associated with fast growth in European domesticated common carps. **Fig. S9.** The TE profile in one-to-one ohnolog pairs associated with high growth rate in European domesticated common carps. **Fig. S10.** SNPs density around one-to-one ohnolog pairs associated with high growth rate in European domesticated common carps.

**Additional file 2:** **Table S1.** Detailed information of samples and genomic data used in this study. **Table S2.** The genome-wide average genetic differentiation (FST) of pairwise common carp strains. **Table S3.** The average genetic diversity in each common carp strain. **Table S4.** Strains used in selective sweep analysis for each domesticated trait. **Table S5.** Genomic regions with selection signatures related to scale reduction in common carp domestication. **Table S6.** Function annotation of the 56 genes in genomic regions with selection signals associated with scale reduction in common carp domestication. **Table S7.** Genomic regions with selection signatures related to skin color variation in common carp domestication. **Table S8.** Function annotation of the 77 genes in genomic regions with selection signals associated with skin color variation in common carp domestication. **Table S9.** RNA-seq data involved in this study. **Table S10.** Expression of the five pair ohnologs related to skin color variation in common carp domestication. **Table S11.** Genomic regions with selection signatures related to high growth rate in common carp domestication. **Table S12.** Function annotation of the 266 genes in genomic regions with selection signals associated with high growth rate in common carp domestication. **Table S13.** Ohnolog pairs in the genomic regions with selection signatures associated with domestication and the whole genome in yxYR, GM, and HB strains. **Table S14.** Statistics of duplicate gene pairs in the selection regions. **Table S15.** Expression of the 121 ohnolog pairs (1:1) between subgenomes associated with high growth rate in European common carps. **Table S16.** TE content in genomic regions related to candidate genes under selection on fast growth in European common carps and their ohnologs in GM genome.

## Data Availability

All data generated or analyzed during this study are included in this published article, supplementary information files, and publicly available repositories. Sequence data generated in this study have been deposited in NCBI Sequence Read Archive (SRA) under the BioProject PRJNA1026856 [60], and accession numbers for all involved sequencing data are listed in Supplementary Tables 1 and 9 [60, 61, 68,62,63,64,65,66,67]. Additional data files were uploaded to Figshare [93].

## Abbreviations

WGD: Whole-genome duplication
QTL: Quantitative trait locus
TE: Transposable element
YRI: Yellow River carp sampled from Hohhot in Inner Mongolia
HLJ: Heilongjiang River carp
AM: American carp
DN: Danube River carp
yxYR14: Yuxuan Yellow River carp
yxYR19: Yuxuan Yellow River carp
HB: Hebao red carp
XG: Xingguo red carp
OJ: Oujiang color carp
KOI: Koi carp
GM: German mirror carp
SP: Songpu mirror carp
SV: Szarvas 22 mirror carp
Π: Nucleotide diversity
*F*_ST_: Pairwise fixation index
LD: Linkage disequilibrium
EHH: Extended haplotype homozygosity
SVC: Spring viremia of carp
*fgfr1a1*: Fibroblast growth factor receptor 1a1
*abca12*: ATP-binding cassette subfamily A member 12
*morc3a*: MORC family CW-type zinc finger 3a
*erbb4b*: Erb-b2 receptor tyrosine kinase 4b
*mst1ra*: Macrophage-stimulating 1 receptor a
*sema3fa*: Sema domain, immunoglobulin domain [Ig], short basic domain, secreted, and [semaphorin] 3Fa
*ip6k2a*: Inositol hexakisphosphate kinase 2a
SNP: Single-nucleotide polymorphism
GATK: Genome Analysis Toolkit
GVCF: Genomic variant call format
*r*^2^: Squared correlation coefficient
PCA: Principal component analysis
SFS: Site frequency spectra
CLR: Composite likelihood ratio
GO: Gene Ontology
KEGG: Kyoto Encyclopedia of Genes and Genomes
TPM: Transcripts per million
EDTA: Extensive de novo TE annotator
