# Supplementary material for: Asymmetric and parallel subgenome selection co-shape common carp domestication
Source: BMC Biol. 2024 Jan 2;22:4. doi: 10.1186/s12915-023-01806-9 (PMC10762839; doi:10.1186/s12915-023-01806-9)
Supplement: Supplementary file 1 — Additional file 1: Fig. S1. Bayesian model–based clustering analysis for 51 common carp individuals. Fig. S2. Population genetic parameter estimation. Fig. S3. Selection signals related to scale reduction. Fig. S4. Haplotype analyses in gene erbb4b and trim33 related to scale reduction. Fig. S5. Selection signals related to vibrant skin color. Fig. S6. Gene expression of the two parallel selected ohnolog pairs. Fig. S7. The putative selection sweep signals related to high growth rate. Fig. S8. Expression divergence between one-to-one ohnologs including genes associated with fast growth in European domesticated common carps. Fig. S9. The TE profile in one-to-one ohnolog pairs associated with high growth rate in European domesticated common carps. Fig. S10. SNPs density around one-to-one ohnolog pairs associated with high growth rate in European domesticated common carps. [file 12915_2023_1806_MOESM1_ESM.docx]

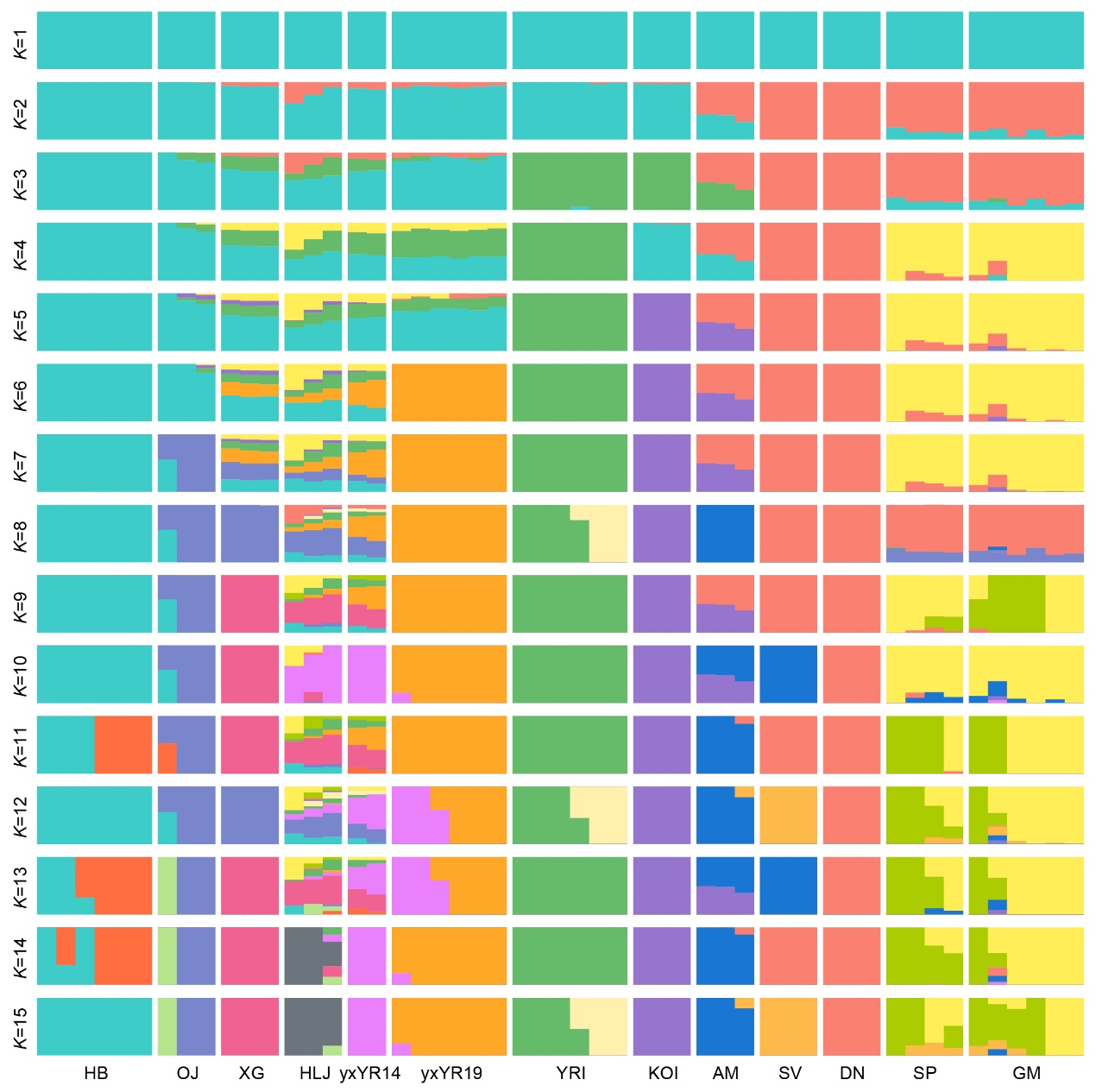


**Fig. S1 Bayesian model-based clustering analysis with different numbers of groups (K = 2–13) for 51 common carp individuals.** Each vertical bar represents one common carp individual, and the x-axis shows different strains. Each color represents one putative ancestral background, and the y-axis quantifies ancestry membership.


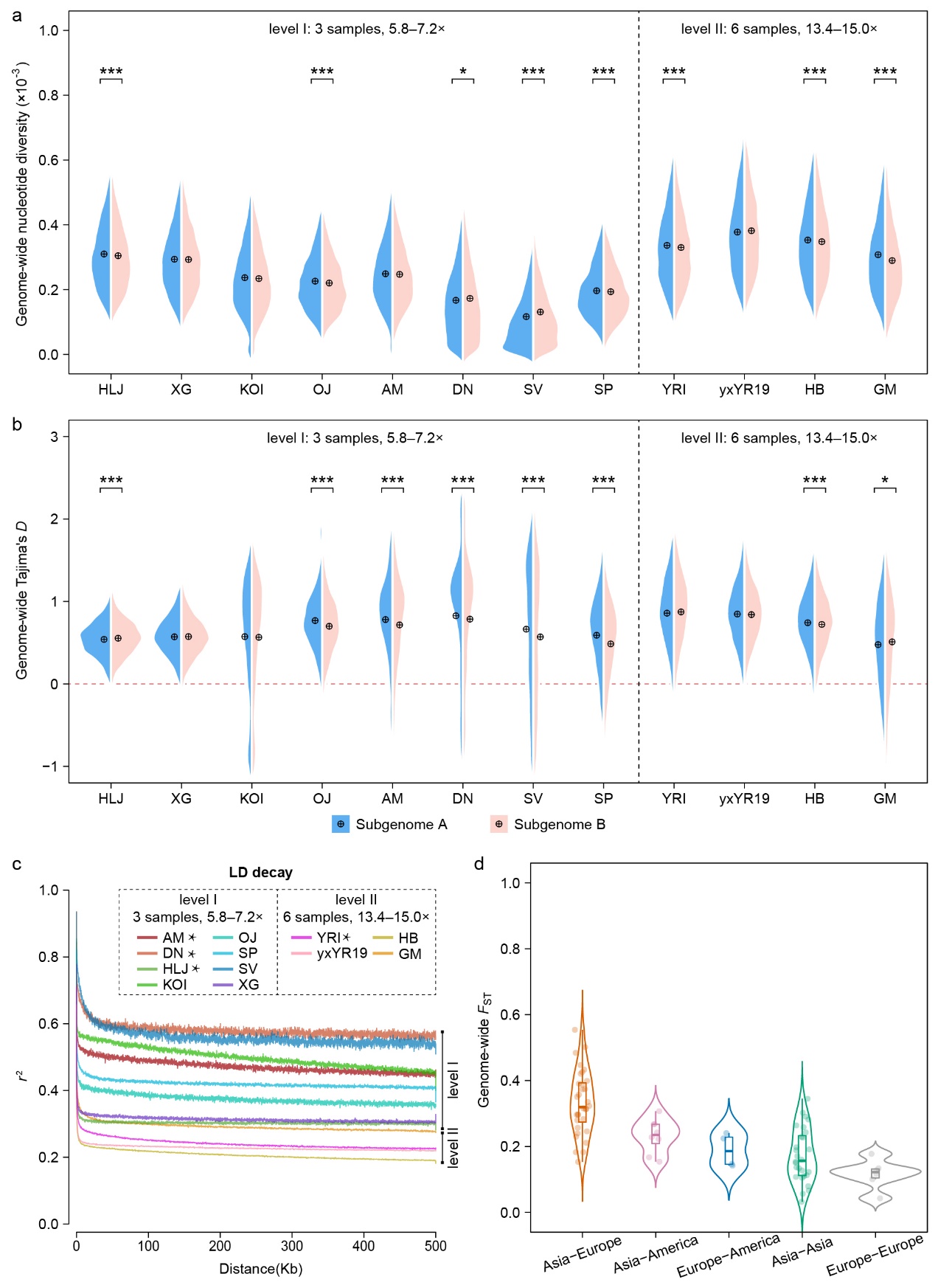


**Fig. S2 Population genetic parameter estimation.** Genome-wide nucleotide diversity (a) and Tajima’s *D* (b) were calculated for populations with three individuals and mean sequencing depth of 5.8–7.2× and populations with six individuals and mean sequencing depth of 13.4–15.0×. The points denote the mean value. (c) The decay of linkage disequilibrium (LD) in common carps. (d) The population divergence (*F*_ST_) between different geographical regions.


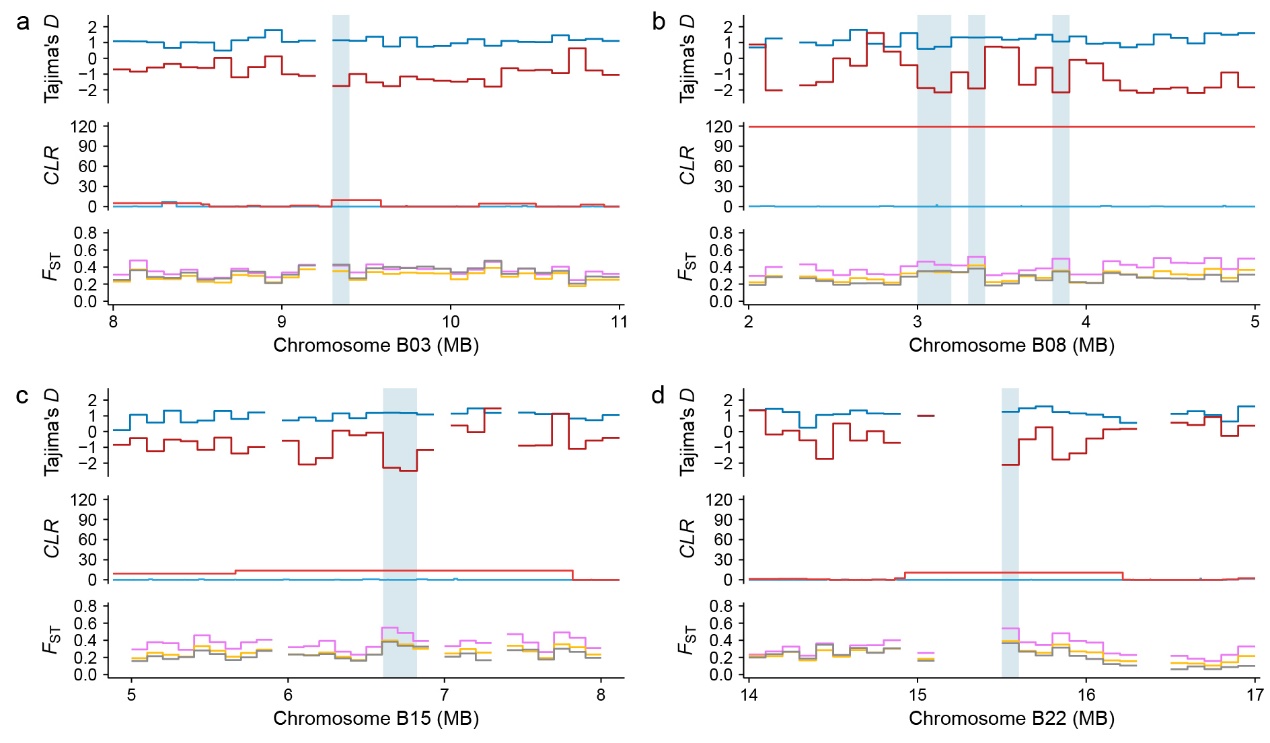


**Fig. S3** **Selection signals related to scale reduction on chromosomes of B03 (a), B08 (b), B15 (c), and B07 (d).** The putative selection sweep signals were identified with *F*_ST_, Tajima’s *D*, and *CLR* score. Tajima’s *D* and *CLR* scores were calculated for the scale-reduced group (red lines) and the fully scaled wild group (blue lines), respectively. *F*_ST_ was calculated between the scale-reduced group and the fully scaled wild group (the grey line), the scale-reduced group and the fully scaled wild and domesticated group (the orange line), and the scale-reduced group and the fully scaled domesticated group (the pink line). Vertical bars with light blue indicate genomic regions under selection.

**
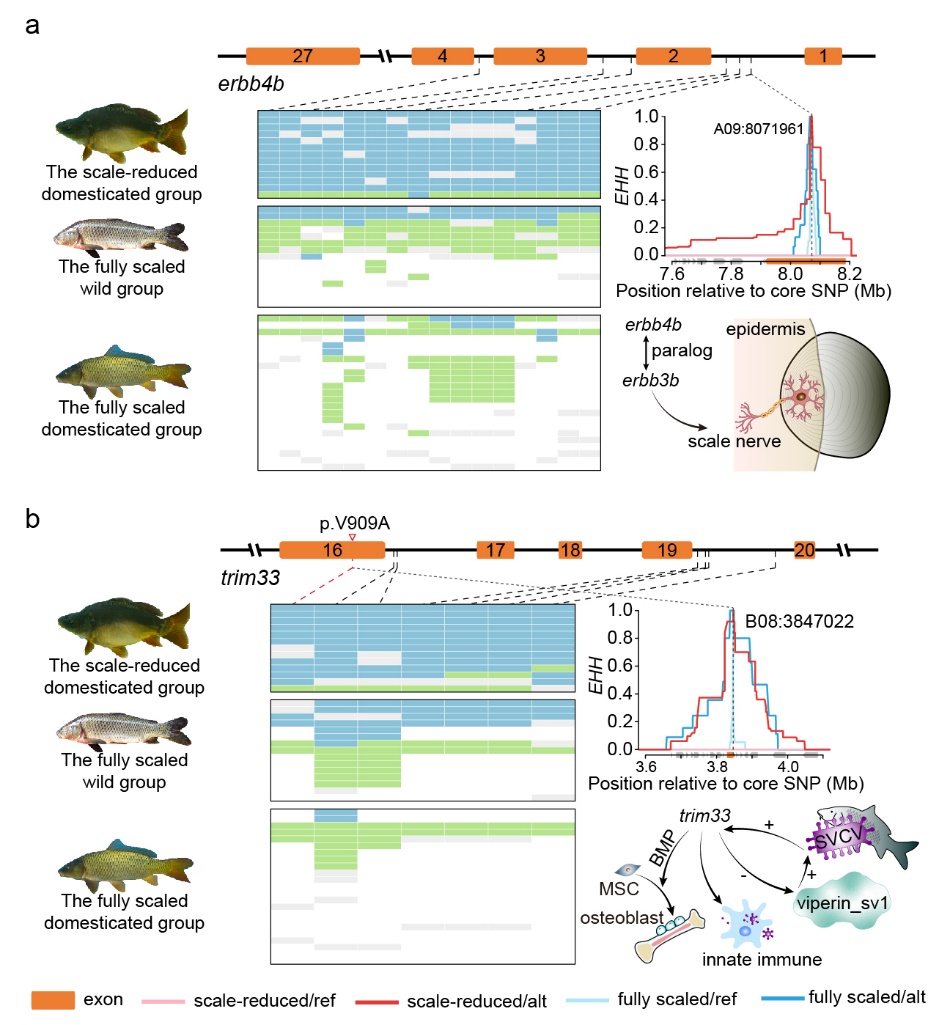
**

**Fig. S4 Haplotype analyses in gene *erbb4b* and *trim33* related to scale reduction.** (a) Genotypes of SNPs in gene *erbb4b* and extended haplotype homozygosity around the crucial SNP “A09:8071961”. (b) Genotypes of SNPs in gene *trim33* and extended haplotype homozygosity around the crucial SNP “B08:3847022”.


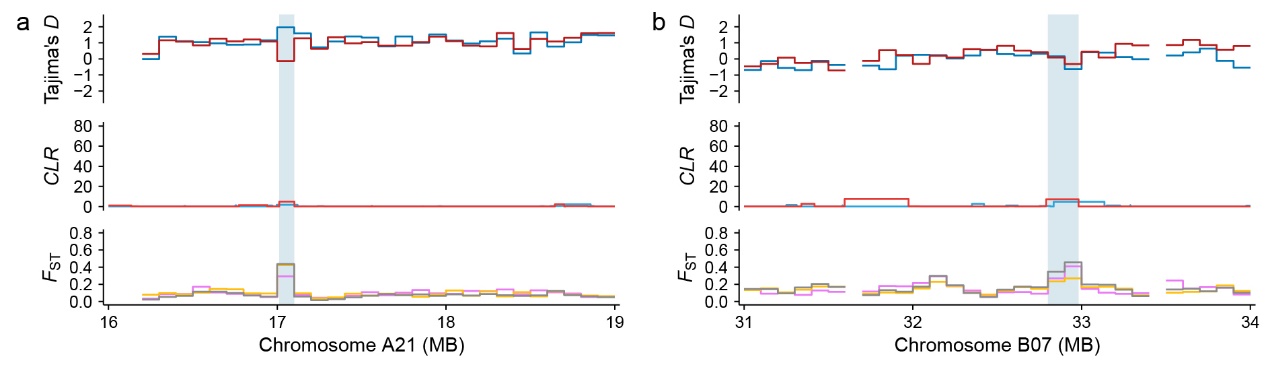


**Fig. S5 Selection signals related to vibrant skin color on chromosomes of A21 (a) and B07 (b).** The putative selection sweep signals on chromosomes were identified with *F*_ST_, Tajima’s *D*, and *CLR* score. Tajima’s *D* and *CLR* scores were calculated for the skin-vibrant group (red lines) and the skin-caesious wild group (blue lines), respectively. *F*_ST_ was calculated between the skin-vibrant group and the skin-caesious wild group (the grey line), the skin-vibrant group and the skin-caesious wild and domesticated group (the orange line), and the skin-vibrant group and the skin-caesious Asian wild group (the pink line). Vertical bars with light blue indicate genomic regions under selection.


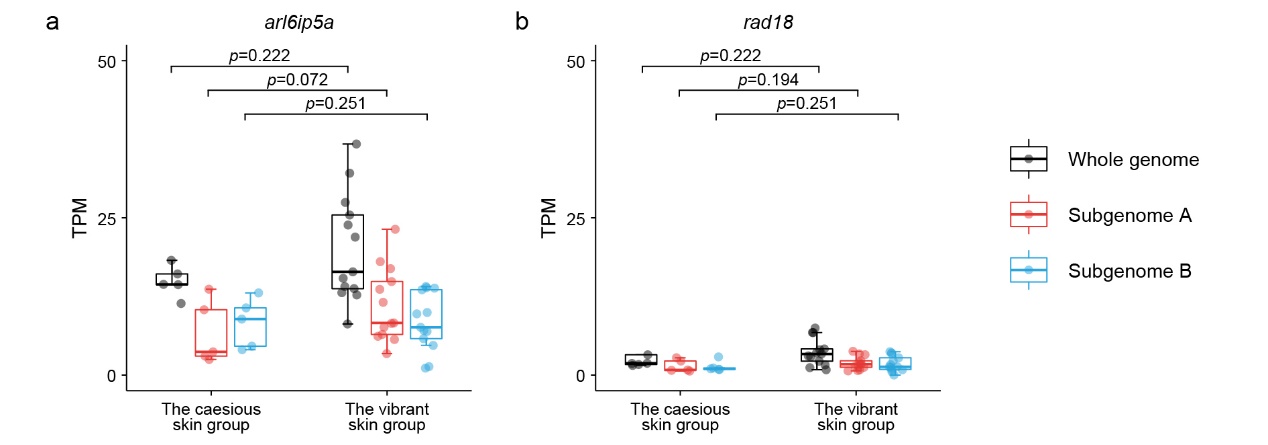


**Fig. S6 Gene expression of the two parallel selected ohnolog pairs.**

(a) Gene *arl6ip5a*. (b) Gene *rad18*.
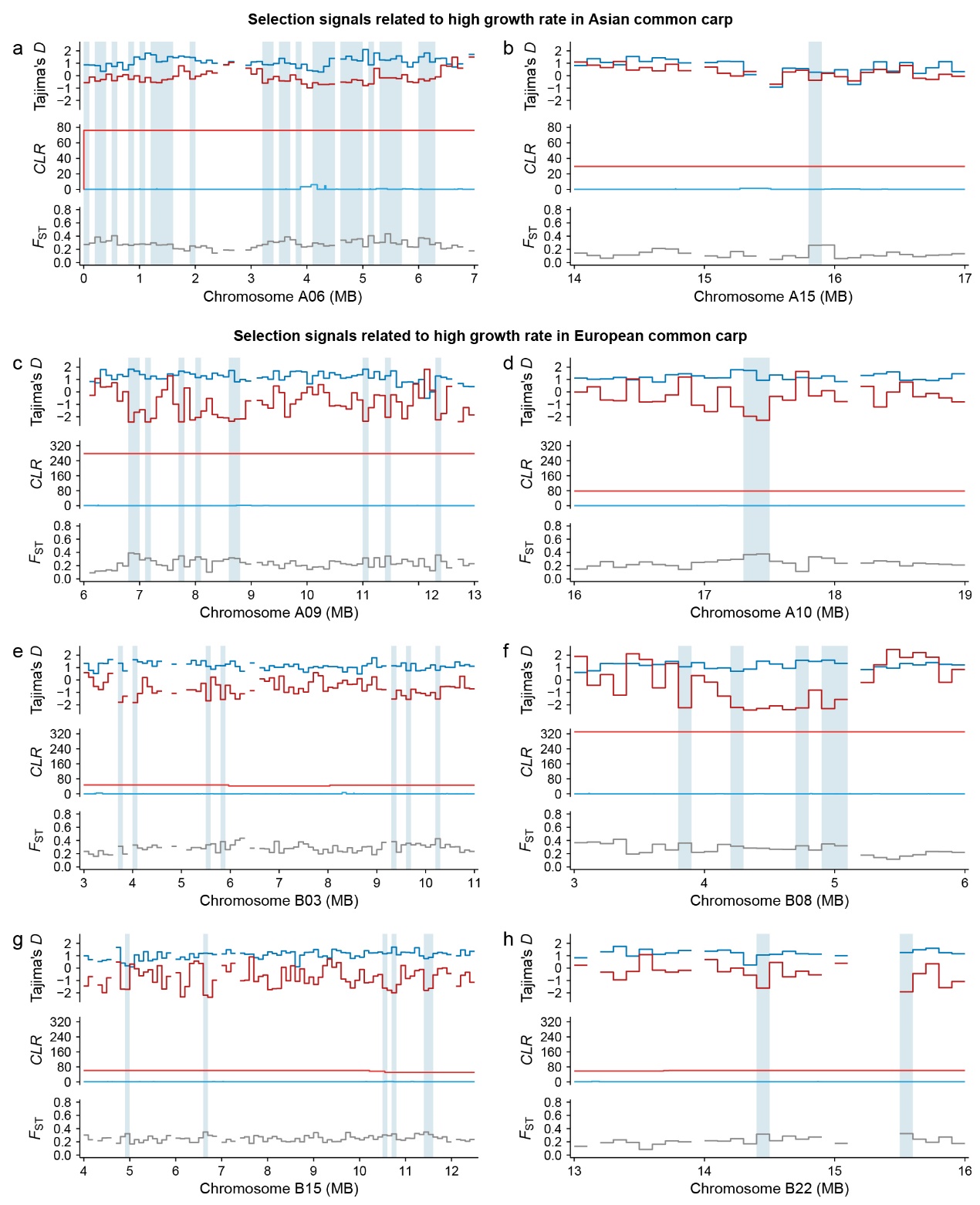


**Fig. S7 The putative selection sweep signals related to high growth rate according to *F*_ST_, Tajima’s *D*, and *CLR* score.** The putative selection sweep signals on chromosomes of A06 (a) and A15 (b) related to high growth rate in Asian domesticated common carps. The putative selection sweep signals on chromosomes of A09 (c), A10 (d), B03 (e), B08 (f), B15 (g), and B22 (h) related to high growth rate in European domesticated common carps. The Tajima’s *D* and *CLR* values were calculated for the high growth rate group (red lines) and the normal growth rate group (blue lines), respectively. *F*_ST_ was calculated between the high growth rate group and the normal growth rate group. Vertical bars with light blue indicate genomic regions under selection.


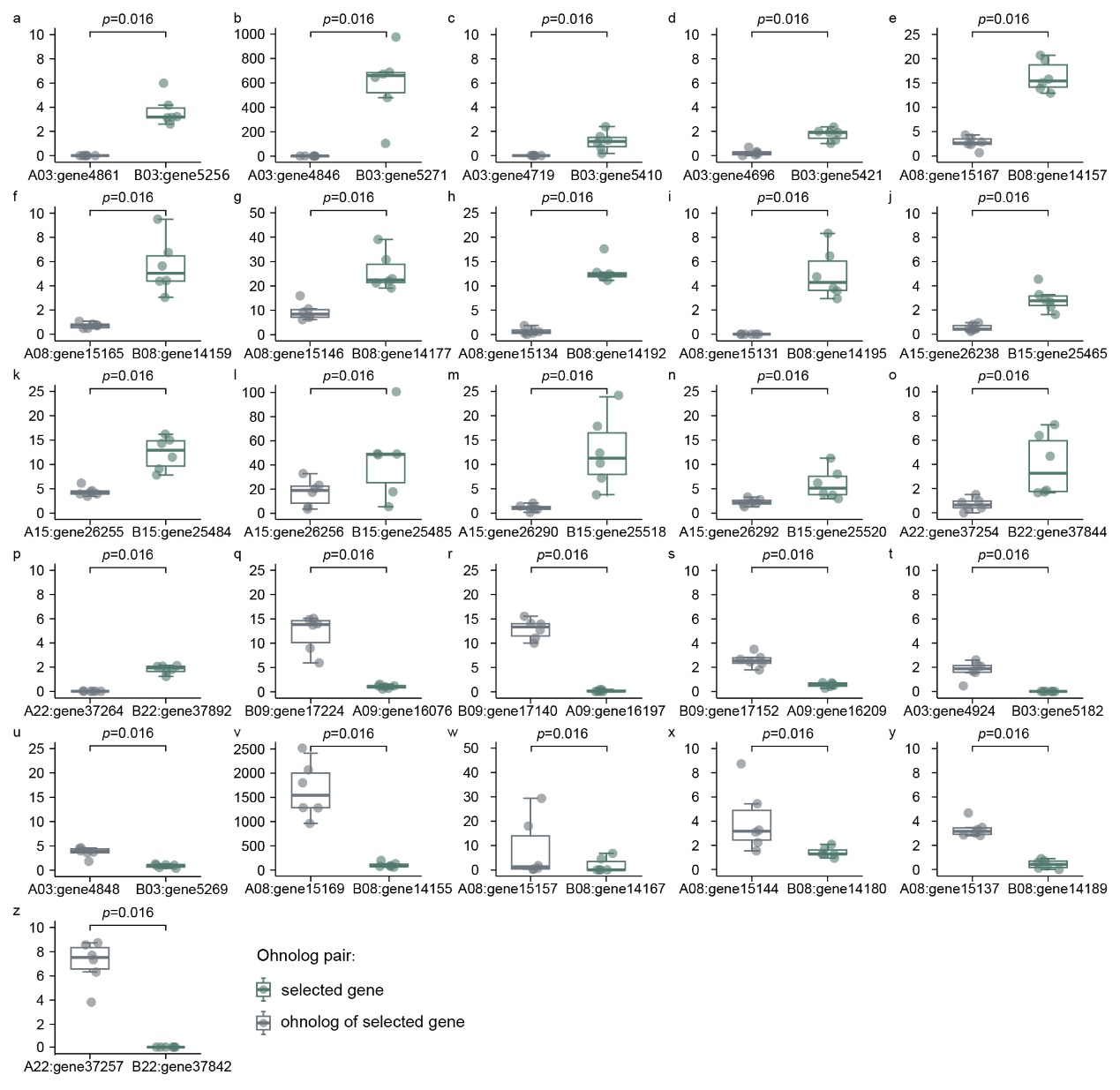


**Fig. S8 Expression divergence between one-to-one ohnologs including genes associated with fast growth in European domesticated common carps.**


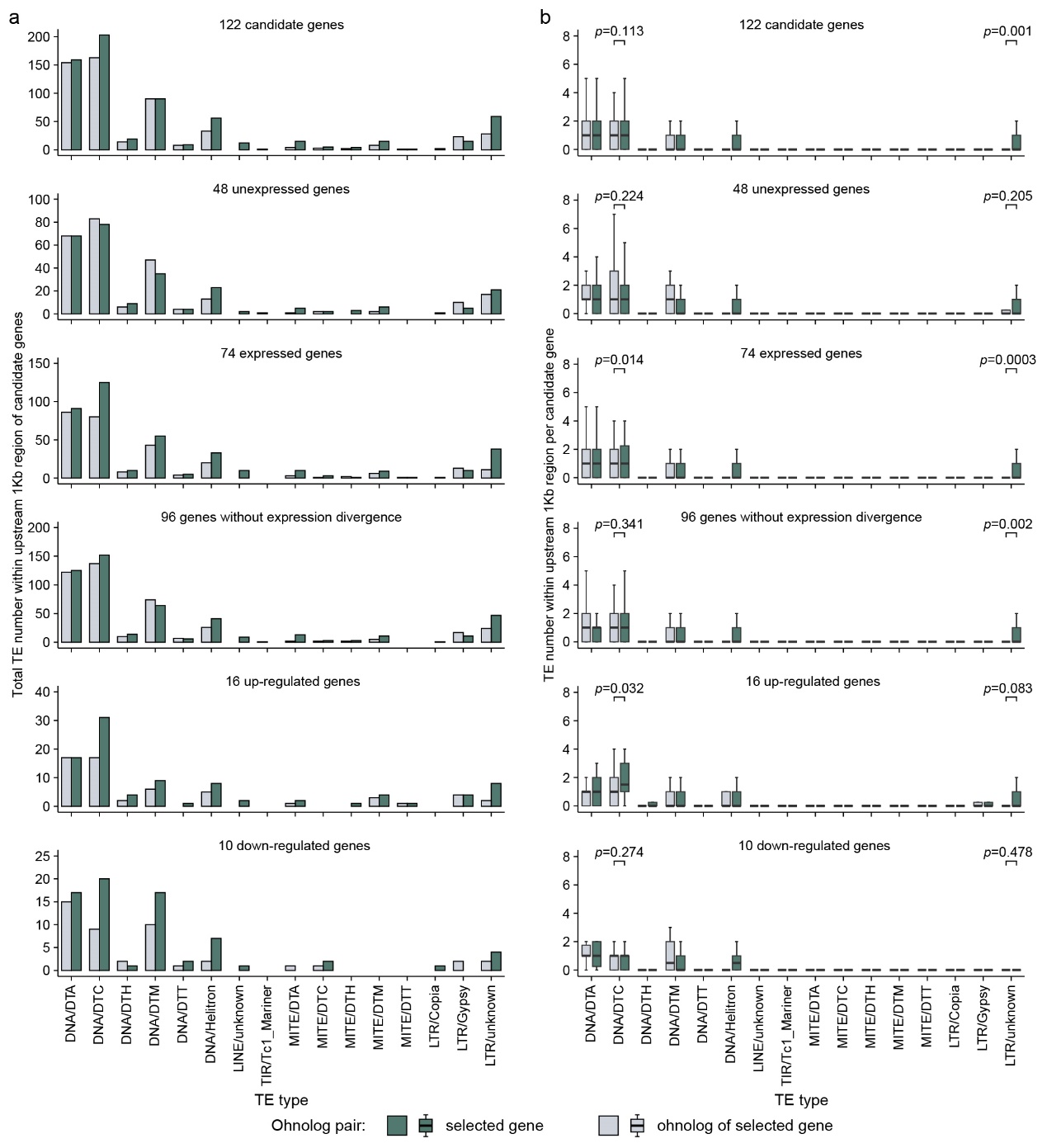
 **Fig. S9** **The TE profile in 122 pairs of one-to-one ohnologs including genes associated with high growth rate in European domesticated common carps in GM genome.** (a) Total TE number within in upstream 1 Kb region of candidate genes; (b) TE number within upstream 1 Kb region in each gene.


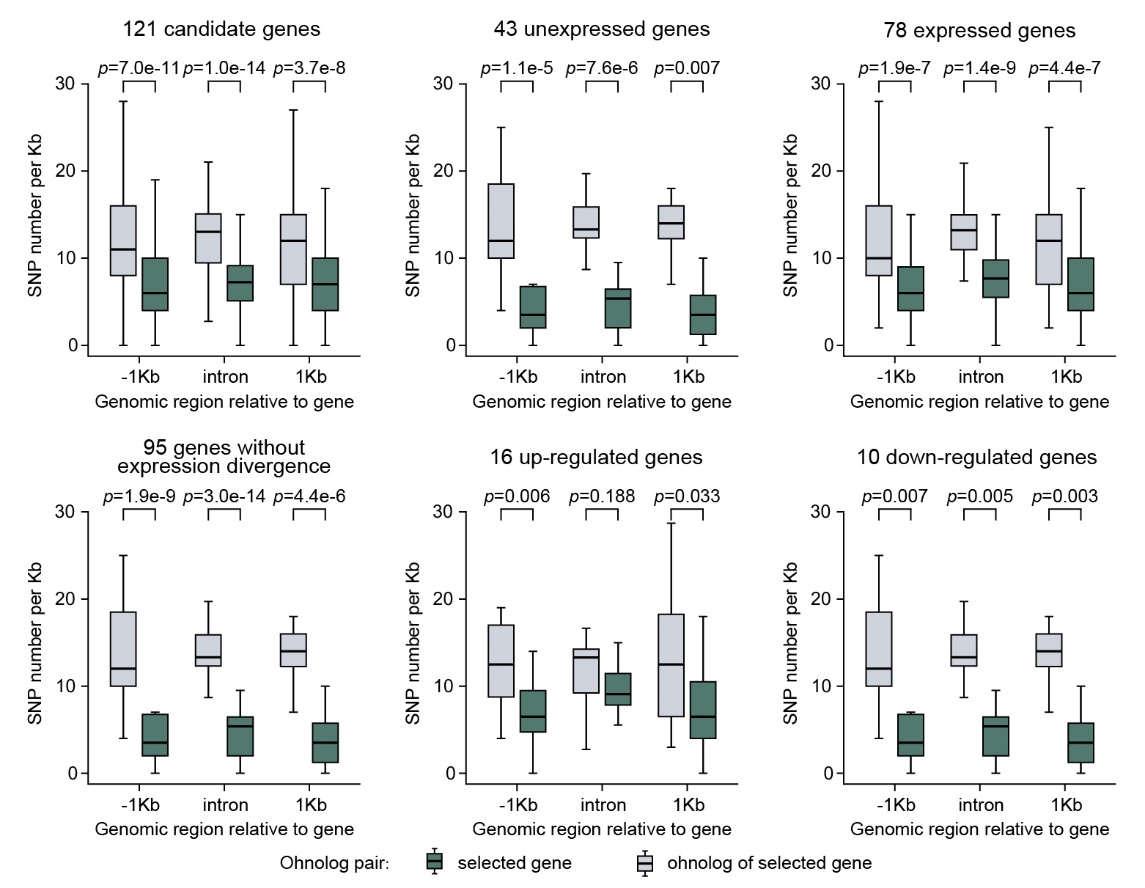


**Fig. S10 SNPs in gene body (intron), upstream (1 Kb) and downstream (1 Kb) regions between the 121 pairs of ohnologs, 43 pairs of expressed ohnologs, 78 pairs of expressed ohnologs, 95 pairs without expression divergence, 16 pairs of ohnologs with twofold up-regulated expression in selected genes, and 10 pairs of ohnologs with twofold down-regulated expression in selected genes, respectively.**
